# Impact of Coffee Intake on Measures of Wellbeing in Mice

**DOI:** 10.3390/nu16172920

**Published:** 2024-09-01

**Authors:** Nuno J. Machado, Ana Paula Ardais, Ana Nunes, Eszter C. Szabó, Vasco Silveirinha, Henrique B. Silva, Manuella P. Kaster, Rodrigo A. Cunha

**Affiliations:** 1 CNC-Center for Neuroscience and Cell Biology, University of Coimbra, 3004-504 Coimbra, Portugal; nuno.machado.br@gmail.com (N.J.M.); ardais@gmail.com (A.P.A.); nunes.ana@gmail.com (A.N.); eszterszaboster@gmail.com (E.C.S.); vasco.silveirinha@gmail.com (V.S.); hbdasilva@gmail.com (H.B.S.); manu.kaster@gmail.com (M.P.K.); 2 Faculty of Medicine, University of Coimbra, 3004-504 Coimbra, Portugal; 3 MIA-Portugal, Multidisciplinary Institute of Aging, University of Coimbra, 3004-504 Coimbra, Portugal

**Keywords:** coffee, caffeine, adenosine receptor, antioxidant, mood, sociability, anhedonia, ventral hippocampus

## Abstract

Coffee intake is increasingly recognized as a life-style factor associated with the preservation of health, but there is still a debate on the relative effects of caffeinated and decaffeinated coffee. We now tested how the regular drinking of caffeinated and decaffeinated coffee for 3 weeks impacted on the behavior of male and female adult mice. Males drinking caffeinated coffee displayed statistically significant lower weight gain, increased sensorimotor coordination, greater motivation in the splash test, more struggling in the forced swimming test, faster onset of nest building, more marble burying and greater sociability. Females drinking caffeinated coffee displayed statistically significant increased hierarchy fighting, greater self-care and motivation in the splash test and faster onset of nest building. A post-hoc two-way ANOVA revealed sex-differences in the effects of caffeinated coffee (*p* values for interaction between the effect of caffeinated coffee and sex) on the hierarchy in the tube test (*p* = 0.044; dominance), in the time socializing (*p* = 0.044) and in the latency to grooming (*p* = 0.048; selfcare), but not in the marble burying test (*p* = 0.089). Intake of decaffeinated coffee was devoid of effects in males and females. Since caffeine targets adenosine receptors, we verified that caffeinated but not decaffeinated coffee intake increased the density of adenosine A_1_ receptors (A_1_R) and increased A_1_R-mediated tonic inhibition of synaptic transmission in the dorsolateral striatum and ventral but not dorsal hippocampus, the effects being more evident in the ventral hippocampus of females and striatum of males. In contrast, caffeinated and decaffeinated coffee both ameliorated the antioxidant status in the frontal cortex. It is concluded that caffeinated coffee increases A_1_R-mediated inhibition in mood-related areas bolstering wellbeing of both males and females, with increased sociability in males and hierarchy struggling and self-care in females.

## 1. Introduction

Coffee is the most widely consumed beverage after water, thanks to its acute psychostimulant effects, bolstering attention, arousal and reaction time to sensory stimuli [1,2], although frequently enhancing anxiety upon consumption of over 4 cups of coffee [3]. Greater interest is now devoted to the effects of regular rather than of acute consumption of moderate doses of coffee since the former decreases the incidence of several chronic diseases [4,5]. Coffee is a mixture of over 2000 potentially bioactive substances with particularly high levels of chlorogenic acids and caffeine [6]. Accordingly, the prophylactic health benefit associated with the regular intake of coffee has mostly been associated with the effects of caffeine [7,8]. At non-toxic doses caffeine selectively acts as an antagonist of adenosine receptors [9,10] to increase allostasis and normalize biological functions [11], namely decreasing the incidence of neuropsychiatric diseases [12]; this involves an epigenetic, transcriptomic and metabonomic preconditioning [13,14] coupled to a reorganization of brain responsiveness [15,16]. Furthermore, other prominent constituents of coffee, namely chlorogenic acids, have also been shown to control neuronal dysfunction [17] as well as chronic diseases such as hypertension, cancer, diabetes or dementia [18], presumably through the ability of chlorogenic acids to bolster the antioxidant potential [19]. This translates into some debate on the relative health benefits of the consumption of regular caffeinated coffee (designated from now on as caff) and of decaffeinated coffee (designated from now on as decaf), which has a low content of caffeine but is as rich in chlorogenic acids as caff [20]. This is best heralded by the conflicting results on the relative effects of caff and decaf intake on the decreased incidence of chronic diseases associated with aging (e.g., [21,22,23]). Moreover, some studies reported sex-related differences in some particular health benefits (e.g., stroke, dementia, Parkinson’s disease) of the regular coffee intake [24], although the increased healthspan on aging is observed both in men and women (e.g., [22,23]). In contrast to these robust evidences linking coffee intake with a lower incidence of chronic diseases, the effects of the regular intake of moderate doses of caff and decaf on the behavior (rather than on disease susceptibility) of healthy animals and humans still remains to be thoughtfully investigated.

The present study carried out a behavioral characterization in male and female mice of the impact of the regular consumption of caff and decaf, which was complemented by a subsequent analysis of the antioxidant status in the frontal cortex and of the adenosine modulation of synaptic transmission in the striatum and in the hippocampus. These brain regions were selected as representative of brain circuits controlling memory (frontal cortex and dorsal hippocampus; [25]), locomotion (striatum, which also processes motivational behaviors; [26,27]) and mood (frontal cortex and ventral hippocampus; [28]).

## 2. Methods

### 2.1. Animals

C57bl\6j mice (10–12 weeks old) were acquired to Charles River (Barcelona, Spain). We used a similar number of males and females (28 of each sex organized in 3 groups of 8 mice each, plus 4 mice for social interaction tests). Mice were then divided into 3 groups (water, caffeinated and decaffeinated coffee intake) according to their hierarchical ranking; this ranking was established using the tube test (see below) where each mouse was tested against the 23 mice of the same sex, attributing to each win a value of 1 and each loss a value of 0. Summing the 23 tests allowed computing an average to define the hierarchy of the group. Mice were then sequentially separated (higher ranking to group 1, second to group 2, third to group 3, fourth to group 1 second cage, fifth to group 2 second cage, etc.) Mice were maintained in groups of four per cage in a temperature-controlled room (22 ± 2 °C) with a 12 h light/12 h dark cycle (lights on at 8:00 a.m.) and with free access to food and to water or the tested beverages. Mice were handled twice daily during the whole period of 25 days before behavioral testing. The study was performed in accordance with the principles and procedures outlined as “3Rs” in the guidelines of the European Union (2010/63/EU), FELASA and ARRIVE and was approved on 30 October 2013 by the Ethics Committee for Animal Research of the Center for Neuroscience and Cell Biology of the University of Coimbra (ORBEA-78-2013). All efforts were made to reduce the number of animals used and to minimize their stress and discomfort. In all manipulations, the experimenters were unaware of the experimental group to which each animal belonged.

### 2.2. Drugs and Treatments

In this naturalistic study, we used aqueous extracts of commercial blends that are popular in Portugal and are representative of caffeinated coffee (caff, Café Moído, Moagem Máquina, Delta Cafés^®^) and of decaffeinated coffee (decaf, Café Moído Torrado Descafeinado Universal, Delta Cafés^®^). Each was prepared daily by dissolving 1 g of powder into 1 L of hot water, followed by a centrifugation at 50× *g* and the supernatants were placed in the drinking bottles supplied to the mouse cages (4 mice per cage). Mice had free access to these coffee-like beverages during 12 h/day, from 8 pm to 8 am (light off period) until their sacrifice. This roughly corresponds to a daily intake of 0.25–0.35 mg of caffeine and 1.2–1.6 mg of chlorogenic acids in the circa 14–15 mL of coffee estimated to be freely consumed daily by mice (14.9 ± 0.3 mL for males and 14.2 ± 0.1 mL for females, *n* = 4), similar to the consumption of decaffeinated coffee (15.0 ± 0.5 mL for males and 13.8 ± 0.2 mL for females, *n* = 4) or of water (15.1 ± 0.7 mL for males and 13.5 ± 0.5 mL for females, *n* = 4). This amount of coffee translates into *circa* 350 mg of caffeine in adult humans [29]. However, these values are approximate since the mice were kept in groups in their home cages during the consumption of the coffees to avoid stress-related isolation; furthermore, in this naturalistic-like study, we did not quantify either the levels of caffeine or of chlorogenic acids neither in the beverages nor in fluids or tissues of the mice.

For the electrophysiological experiments, the adenosine A_1_ receptor (A_1_R) agonist N^6^-cyclopentyladenosine (CPA) and the A_1_R antagonist 8-cyclopentyl-1,3-dipropylxanthine (DPCPX, both from Sigma, Sintra, Portugal) were prepared as 5 mM stock solutions in dimethylsulfoxide and a dilution was prepared in the superfusion medium, controlling for the impact of the residual amount of dimethylsulfoxide. CPA was applied in 4 increasing concentrations: 6, 10, 30 and 60 nM, as previously done to estimate the potency and efficacy of A_1_R [30,31] and DPCPX (50 nM) was used at a supramaximal but selective concentration to prevent the activation of A_1_R by endogenous adenosine [31,32].

### 2.3. Behavioral Testing

**Figure 1:**
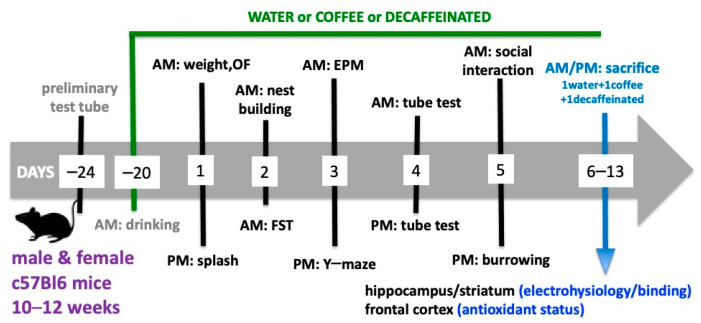
**Overall schematic presentation of the experimental protocol.** OF: open field test; FST: forced swimming test; EPM: elevated plus maze.

The mice were first weighted 21 days after beginning their controlled intake of water or coffees, before starting the behavioral tests on day 1. We used a tight schedule of behavioral characterization, with a minimal time interval between each test required to avoid cross-interference between the tests [14,33,34]. All behavior tests were carried out from 8 AM until 7 PM in the 22nd until the 27th days after beginning the supply of caffeinated and decaffeinated coffee, according to the schedule presented in Figure 1. The tests were carried out by experimenters who were unaware of the beverages consumed, in a sound-attenuated room with an 8 lux illumination and visual cues on the walls, to which the animals were previously habituated. The apparatuses were cleaned with 10% ethyl alcohol to remove any odors after testing each animal or groups of animals.

Open field: Locomotion and exploratory behavior were monitored in the morning of day 1, using an open-field arena, where each mouse was placed in the center of the open field to record during 10 min their total locomotion (peripheral locomotion and central locomotion, the later used as surrogate indicator of anxiety) and the number of rearing events as a proxy of sensorimotor coordination [33].

Splash test: The splash test was carried out as previously described [33,35] on the afternoon of day 1, by squirting a 10% sucrose solution on the dorsal coat of the animal and measuring the onset of grooming bouts (head washing and nose/face and body grooming) as an estimation of self-care and the time spent grooming (as a measure of motivation) over 5 min.

Nest building: The motivation to engage in nest building activity was evaluated as previously described as an indicator of wellbeing [36] on the morning of day 2. A pre-test habituation session was first carried out where the mouse was placed individually in a new cage with some bedding (circa 20%) from the home cage and was supplied with a single cotton nestlet with cotton fiber nestlets (5 cm × 5 cm, 5 mm thick, ~2.5 g). After 90 min, the nest was removed, a new nestlet was placed in the center of the cage and we monitored the time until beginning the construction of a new nest (latency to nest building). After 90 min, the mouse was returned to its home cage.

Forced swimming test: The depressive-like behavior was evaluated in the forced swimming test, carried out in the afternoon of day 2, as previously described [33,34]. Mice were placed in individual glass cylinders (40 cm in height and 17 cm in diameter) containing water (water depth was 30 cm, kept at 25 ± 1 °C) to measure the total duration of immobility and climbing attempts during a 10-min session. A mouse was regarded as immobile when floating motionless or making only those movements necessary to keep its head above the water. The climbing (or struggling) behavior was defined as upward-directed movements of the forepaws usually along the walls of the swimming cylinder. Diving and face shaking behaviors were not considered.

Elevated plus maze: Anxiety was further assessed in the morning of day 3, using the elevated-plus maze, where each animal was allowed to explore during 8 min the open and closed arms of the crossed maze (central platform with 6 × 6 cm connecting 4 crossed arms 18 cm long and 6 cm wide with 2 opposite closed arms surrounded by walls 6 cm high and 2 opposite open arms devoid of enclosing walls). The number of entries in both open and closed arms were recorded, considering an entry only when the whole body and four paws were inside an arm [33,34].

Y-maze: Working memory was assessed in a spontaneous alternation paradigm using the Y-maze test on the afternoon of day 3, as previously described [37,38]. Individual mice were placed at the end of one arm and allowed to freely explore the maze for 5 min. The sequence of entrances in each arm was recorded, and the number of alternations (sequential entrance in the three different arms) was quantified. The percentage of spontaneous alternation consists in the percentage of alternations of the total possible number of alternations (total number of arm changes—2).

Tube test: Dominance was assessed in the tube test on day 4. We used transparent Plexiglas tubes with a 30-cm length and a 3-cm internal diameter, both for habituation and for the tests, as previously described [39]. Habituation was done in 2 steps. First two tubes were placed in each cage with 4 mice for 2 h. Then each mouse was individually placed at one end of a tube with a palatable food pellet placed at the exit of the other end of the tube and this was repeated 4 times in both directions. Two hours after, the tests were carried out by holding the two tested mice by their tails inside each end of the tube and releasing them simultaneously. Winning was established when one of the mice retreated the other to a distance equivalent to the length of its body. The test was carried out at two different time points: 4 days before starting any treatment to rank all the animals (males were tested versus all other males and females versus all other females) to separate them in ‘balanced groups’ (see above under ‘Animals’) and on day 4 of the behavioral tests, i.e., 25 days after free intake of water, caff or decaf. In this second test period, each mouse under one treatment was only tested against all mice under the other treatments. We ranked a winning as ‘1’ and a loss as ‘0’ and computed the average for each mouse. 

Sociability test: The social testing apparatus used in the morning of day 5, was a rectangular, three-chambered box with each chamber measuring 20 × 40 × 25 cm, with rectangular openings (4 × 4 cm in diameter) allowing access into each chamber. The test mouse was first placed in the middle chamber and allowed to explore the 3 chambers for 5 min. An unfamiliar male or female C57BL/6J mouse (of the same age) that had no prior contact with the tested mouse, was placed inside a gridded cylinder, which allowed nose contact between the bars, but prevented fighting, in one of the side chambers. After 10 min, the tested mouse was allowed to explore the entire social test box during 10 min to record the time spent interacting with the encaged unfamiliar mouse, as previously described [40].

Marble burying test: The burrowing behavior, which can be considered an indicator of wellbeing [41], was evaluated on the afternoon of day 5. Each mouse first spent 15 min inside the test cage with fresh bedding. After 15 min, they were again placed in the test cage now containing 3 parallel rows each with 5 glass marbles with 1.2 cm diameter laid over the bedding. Mice were left in the cage during 15 min and the number of buried marbles (more than 2/3 of their volume) was counted, as previously described [42].

### 2.4. Sacrifice of the Animals

Mice were sacrificed in trios of the same sex (1 control drinking only water, 1 from the coffee-drinking group and 1 from the decaffeinated-drinking group) starting the day after terminating the behavioral characterization, during 8 successive days (e.g., 1 trio of males in the morning and 1 trio of females in the afternoon). Sacrifice was carried out by decapitation after deep halothane anesthesia (no reaction to pitching their tail while breathing). After sacrifice, the brain was quickly placed in ice-cold and oxygenated (95% O_2_, 5% CO_2_) artificial cerebrospinal fluid (ACSF; in mM: 124.0 NaCl, 4.4 KCl, 1.0 Na_2_HPO_4_, 25.0 NaHCO_3_, 2.0 CaCl_2_, 1.0 MgCl_2_, 10.0 glucose). The two hemispheres were separated and the two frontal cortices were dissected and pulled together for analysis of the antioxidant status. One hemisphere was used to dissect the hippocampus to prepare slices for electrophysiology and to dissect the striatum to prepare membranes for the binding assay. The other hemisphere was used to prepare coronal slices at the level of the striatum for electrophysiology recordings and to dissect the hippocampus and separate its dorsal and ventral poles to prepare total membranes.

### 2.5. Antioxidant Potential

The frontal cortex was gently homogenized in an ice-cold buffer containing 100 mM NaCl and 20 mM HEPES, pH 7.4, and the homogenate was centrifuged at 3000× *g*. The supernatant was collected and used for different biochemical assays after determining the amount of protein using the bicinchoninic acid (BCA) assay (BCA Protein Assay Kit #A55861 or #23235, Pierce, Thermo Scientific, Porto Salvo, Portugal).

Lipid peroxidation: The estimation of the levels of malondialdehyde (MDA) was done using a spectrophometric assay based on the reactivity of 1-methyl-2-phenylindole with MDA and 4-hydroxyalkenals [43]. Briefly, 325 µL of *N*-methyl-2-phenylindol (10 mM in 3:1 acetonitrile:methanol solution; from Fischer Scientific, Porto Salvo, Portugal) were added to 100 µL of supernatant (or MDA standards with 5 concentrations ranging from 0.1–5 µM, prepared from a 1 mM MDA stock solution in dimethylsulfoxide) and 75 µL of methanesulfonic acid (37%). The mixture was incubated at 45 °C for 20 min before absorbance measurement at 570 nm. A blank (aldehyde replaced by water) was subtracted and data was normalized per mg of protein.

Protein oxidation: We quantified the carbonyl content of proteins as a proxy of protein oxidation, based on their reaction with 2,4-dinitrophenylhydrazine to generate dinitrophenylhydrazones prone to be quantified spectrophometrically [44]. Briefly, a fraction of the cerebral cortex supernatant (of the homogenate centrifuged at 3000× *g*, as described above) was centrifuged at 60,000× *g* during 30 min to remove membranes. The collected supernatant was first used to quantify protein using the micro BCA assay and it was then precipitated with 10% (*w*/*v*) trichloroacetic acid. The pellet resuspended in 0.2% (*w*/*v*) dinitrophenylhydrazine (Sigma) in 2 N HCI and the mixture was incubated at 25 °C during 15 min with continuous stirring and then re-precipitated in 10% (*w*/*v*) trichloroacetic acid. The pellet was resuspended into 6 M guanidine HCl with 20 mM sodium phosphate buffer, pH 6.5, centrifuged at 10,000× *g* for 2 min and the absorbance of the supernatant at 450 nm was used to estimate the amount of carbonylated proteins, expressed in nmol/mg of protein, using an extinction coefficient of 21.0 mM^−1^ × cm^−1^ [44].

Superoxide dismutase: The activity of superoxide dismutase (SOD) was determined by following the reduction of nitroblue tetrazolium at 550 nm in a mixture composed of the supernatant of frontocortical extracts (100–150 μg protein) with 5 μM nitroblue tetrazolium (Sigma), 10 μM hypoxanthine, 5% Triton X-100 in phosphate buffer (50 mM K_2_HPO_4_ and 100 μM EDTA, pH 7.8), during 3 min at 25 °C upon addition of 2 μL xanthine oxidase (0.025 U/mL; from Sigma). The activity of SOD was estimated using a standard curve with known amounts of SOD (0.25–2 U; from Sigma) upon subtraction of a blank without hypoxanthine [45].

Glutathione levels: A fluorimetric assay was employed to determine the levels of reduced glutathione (GSH) in the frontal cortex [46]. Briefly, a fraction of the cerebral cortex supernatant was centrifuged at 60,000× *g* during 30 min and 100 μL of the resulting supernatant was incubated with 100 μL of o-phthalaldehyde (1 mg/mL in methanol; from Sigma) during 15 min at room temperature in 20 volumes of phosphate buffer before determining the fluorescence at 420 nm upon excitation at 350 nm. The GSH levels were determined by comparison with 5 standards of GSH (Sigma) ranging from 0.5–10 µM. We did not quantify GSSG and did not compute the GSH/GSSG ratio, which is a more precise level of oxidative status.

Glutathione peroxidase and glutathione reductase activities: The activity of glutathione peroxidase was measured upon pre-incubation during 5 min at room temperature of a frontocortical supernatant sample with 1 mM GSH and 1 U/mL of glutathione reductase (Sigma) in phosphate buffer and monitoring during 5 min, with continuous stirring, the absorbance at 340 nm upon addition of NADPH (250 μM; from Sigma) and tert-butyl hydroperoxide (1.2 mM; from Sigma) [47]. Values from blanks prepared in the absence of NADPH were subtracted. The activity of glutathione reductase was measured upon pre-incubation during 1 min at room temperature of a frontocortical supernatant sample with 250 μM NADPH in phosphate buffer and monitoring during 5 min, with continuous stirring, the absorbance at 340 nm upon addition of oxidized glutathione (2 mM; from Sigma). Values from blanks prepared in the absence of oxidized glutathione were subtracted.

### 2.6. Binding to A_1_ Receptors

To estimate alterations of the density of adenosine A_1_ receptors (A_1_R), we carried out A_1_R binding assays using a supramaximal but selective concentration (2 nM) of a radiolabelled A_1_R antagonist ^3^H-DPCPX, as previously described [30,48]. Briefly, total membranes from the dorsal or ventral hippocampus or from the striatum were prepared by homogenizing the fresh tissue in sucrose solution (0.32 M) containing 50 mM Tris, 2 mM EGTA and 1 mM dithiotreitol, pH 7.6. The resulting homogenates were centrifuged at 1000× *g* for 10 min at 4 °C. The supernatants were transferred to new tubes and centrifuged at 24,000× *g* for 20 min at 4 °C. The pellets were then resuspended in a solution containing 50 mM Tris (pH 7.4 at 23 °C), 2 mM EGTA, 1 mM EDTA and 4 U/mL of adenosine deaminase (from Roche, Amadora, Portugal) and incubated for 30 min at 37 °C to remove endogenous adenosine. The mixture was centrifuged at 24,000× *g* for 10 min at 4 °C, and the pellets resuspended in the incubation solution containing 50 mM Tris (pH 7.4) and 2 mM MgCI_2_. After measuring the protein content, 4 U/mL of adenosine deaminase were added. For the binding assays, carried out in triplicate, this protein suspension (88–123 μg of protein) was incubated for 2 h at room temperature with 2 nM of ^3^H-DPCPX (specific activity of 102.1 Ci/mmol; from DuPont NEN, Boston, MA, USA) in a buffer containing 50 mM Tris, 1 mM EDTA, 2 mM EGTA, pH 7.4, with adenosine deaminase (4 U/mL) before filtration through Whatman GF/C filters (Millipore, Darmstadt, Germany). After drying, the filters were dissolved into scintillation liquid (Ready Safe, Pharmacia, Uppsala, Sweden) and radioactivity counted in a TRICARB 2900TR liquid scintillation analyzer (Perkin Elmer, Lisbon, Portugal), as previously described [30,48]. Results are expressed as specific binding, determined by subtraction of the non-specific binding, which was measured in the presence of 2 mM 8-{4-[(2-aminoethyl)amino]carbonylmethyloxyphenyl}xanthine (XAC, an adenosine receptor antagonist; from Tocris, Bristol, UK) and normalized per amount of protein.

### 2.7. Slice Electrophysiology

One hemisphere was used to prepare coronal slices (400 µm-thick) at the level of the striatum using a Leica VT1200S vibratome (Leica Biosystems, Nußloch, Germany). In parallel, the hippocampus isolated from the other hemisphere was cut perpendicular to its long axis into 400 µm-thick slices using a McIlwain tissue chopper (Brinkmann Instruments, Riverview, FL, USA), allowing to separate slices from the dorsal and from the ventral hippocampus, as previously described (see [49]). Slices were allowed to recover at 32–34 °C for at least 1 h prior to recording, when they were transferred to a submerged recording chamber and superfused at 3 mL/min with oxygenated ACSF kept at 30.8 °C.

The configuration of the extracellular recordings was as previously described [49,50,51]: in slices from the ventral or dorsal hippocampus, the stimulating bipolar concentric electrode was placed in the proximal CA1 *stratum radiatum* for stimulation of the Schaffer collateral fibers and the recording electrode, filled with 4 M NaCl (2–5 MΩ resistance), was placed in the CA1 *stratum radiatum* targeting the distal dendrites of pyramidal neurons to record the evoked field excitatory postsynaptic potential (fEPSP; [32,49]); in the dorsolateral striatum, the stimulating electrode was placed in the external capsule in the white matter above the dorsolateral striatum and the recording electrode, filled with 4 M NaCl (2–5 MΩ resistance), was placed in the superficial layers of the dorsolateral striatum to record the evoked population spike (PS) responses [50,51]. Stimulation was performed using either a Grass S44 or a Grass S48 square pulse stimulator (Grass Technologies, West Warwick, RI, USA) or a Digitimer DS3 stimulator (Digitimer Ltd., Welwyn Garden City, UK), with rectangular pulses of 0.1 ms applied every 20 s. After amplification (ISO-80, World Precision Instruments, Friedberg, Germany; or AxoPatch 200B amplifier, Axon Instruments, Molecular Devices, San José, CA, USA), the recordings were digitized (PCI-6221 acquisition board, National Instruments, Austin, TX, USA; or Digidata 1322A, Axon Instruments), averaged in groups of 8, and analyzed using the WinLTP version 2.10 software [52]. After obtaining a robust response, we first carried out input/output curves in which the fEPSP slope or PS amplitude were plotted versus the stimulus intensity. This allowed selecting an intensity of stimulation between 40–50% of maximal fEPSP (in the dorsal or ventral hippocampus) or PS response (in corticostriatal synapses) to begin testing the effects of the A_1_R agonist CPA and then of the A_1_R antagonist DPCPX. The tested drugs were applied through the superfusion solution and alterations of synaptic transmission were quantified as the % modification of the average value of the fEPSP slope or the PS amplitude taken from 15 to 21 min after beginning the application of each CPA concentrations, in relation to the average value of the fEPSP slope or PS amplitude during the 8 min that preceded application. Likewise, for DPCPX, we compared fEPSP slope or PS amplitude values in the 8 min that preceded drug application in relation to the average values of the 15 to 21 min after its exposure.

### 2.8. Statistical Analysis

The values presented are mean ± S.E.M. with the number of determinations (*n* = 8, preparations from different mice, except if otherwise defined). The comparison of two experimental conditions and control was performed using a one-way analysis of variance (ANOVA), followed by a Tukey’s post hoc multiple comparison test. A post-hoc two-way ANOVA was used to estimate putative interactions between the effects of caffeinated coffee and sex. Statistical significance was considered at *p* < 0.05. Statistical analysis was performed using GraphPad Prism software (version 6, GraphPad Software).

## 3. Results

### 3.1. Behavioral Alterations

The multi-dimensional analysis of behavioral performance of adult mice consuming caffeinated coffee (caff) or decaffeinated coffee (decaf) during at least 3 weeks, revealed that caff triggered some sex-dependent differences, whereas decaf did not alter behavioral performance in both sexes. The behavioral alterations were first analyzed independently in each sex and a post-hoc analysis was then carried to compare sex-dependent alterations.

**Figure 2:**
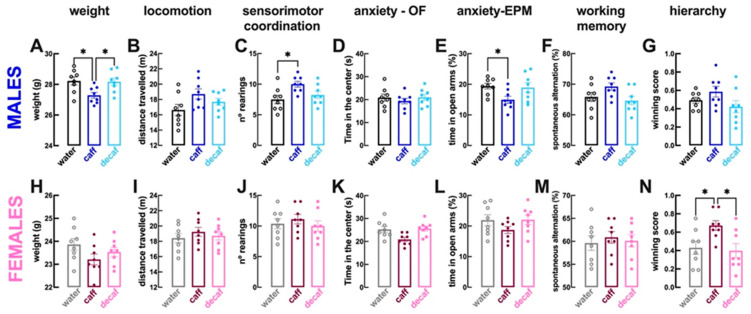
**Caffeinated but not decaffeinated coffee alters behavioral features in both male and female mice**. Adult mice (12 weeks old) either males (upper row in blue) or females (bottom row in pink) had free access to either water (black and grey bars), caffeinated coffee (caff, 1 g/L; in dark blue or dark pink) or decaffeinated coffee (decaf, 1 g/L; in light blue or pink) during the night period for at least 3 weeks before beginning behavioral analysis as described in Figure 1. Compared to control water-consuming mice, caff decreased body weight in males (**A**) but not in females (**H**), did not alter locomotion (distance travelled in the open field test, (**B**,**I**)) or working memory (spontaneous alternations in the Y-maze test, (**F**,**M**)) in either sex, increased sensorimotor coordination (number of rearings in the open field test) in males (**C**) but not in females (**J**) and increased dominance (number of wins in the tube test against mice of the same sex) in females (**N**) but not in males (**G**). Anxiety scores were not altered in the open field test (time in the more aversive central region of the open field, (**D**,**K**)) and an axiogenic effect of caffeinated coffee was observed in the elevated plus maze in males (**E**) but not in females (**L**). The intake of decaf was devoid of effects compared to controls in either sex. Data are mean ± SEM; *n* = 8 mice per group. * *p* < 0.05 between indicated groups using a one-way ANOVA followed by a Tukey’s multiple comparison test.

**Figure 3:**
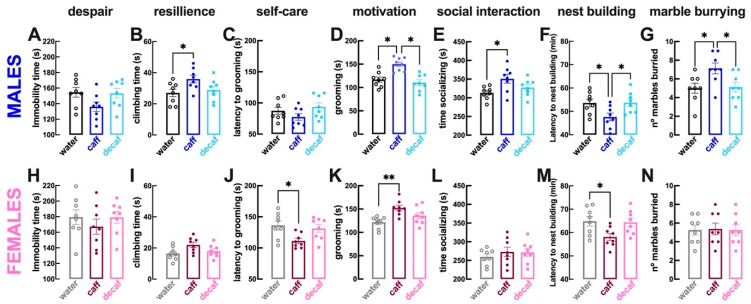
**Caffeinated but not decaffeinated coffee ameliorates mood and wellbeing in both male and female mice**. Adult mice (12 weeks old) either males (upper row in blue) or females (bottom row in pink) had free access to either water (black and grey bars), caffeinated coffee (caff, 1 g/L; in dark blue or dark pink) or decaffeinated coffee (decaf, 1 g/L; in light blue or pink) during the night period for at 3 weeks before beginning behavioral analysis as described in Figure 1. Compared to control water-consuming mice, caff did not alter immobility in the forced swimming test in males (**A**) or females (**H**) but increase resilience (climbing time in the forced swimming test) in males (**B**) but not in females (**I**), increase selfcare (latency to grooming in the splash test) in females (**J**) but not in males (**C**), increased motivation (time spent grooming in the splash test) in both males (**D**) and females (**K**), increased sociability in males (**E**) but not in females (**L**) and increased wellbeing in males and females, as judged by the lower latency to begin building a nest in both males (**F**) and females (**M**) and the number of marbles buried in males (**G**) but not in females (**N**). The intake of decaf in either sex was devoid of effects compared to controls. Data are mean ± SEM; *n* = 8 mice per group. * *p* < 0.05, ** *p* < 0.01 between indicated groups using a one-way ANOVA followed by a Tukey’s multiple comparison test.

Compared to male mice consuming only water (*n* = 8 per group), male mice with access to caff displayed a lower weight (coffee: 27.3 ± 0.19 g vs. water: 28.2 ± 0.27 g; *p* > 0.001; Figure 2A), no alteration of locomotion (*p* = 0.073; Figure 2B) but increased sensorimotor coordination (i.e., number of rearing events; *p* = 0.017; Figure 2C) with no alteration of the time spent in the more aversive central region of the open field (*p* = 0.715; Figure 2D), although a decreased time spent in the open arms of the elevated plus maze suggested an increased anxiety (*p* = 0.049; Figure 2E). Male mice consuming caff had a preserved working memory performance in the Y-maze (*p* = 0.205; Figure 2F), a preserved hierarchy in the tube test (*p* = 0.453; Figure 2G) and a preserved despair behavior in the forced swimming test (*p* = 0.107; Figure 3A), although with apparently more resilience to adversity (increased struggling time, *p* = 0.044; Figure 3B); the tendency for selfcare in the splash test was unaltered (*p* = 0.467; Figure 3C), but motivation was increased (increased grooming time, *p* = 0.001; Figure 3D), as well as sociability (*p* = 0.018; Figure 3E). Finally, caff intake seemed to increase wellbeing of male mice, as gauged by the lower latency to begin nest building (*p* = 0.044 vs. water, *n* = 8; Figure 3F) and the increased burrowing in the marble burying test (*p* = 0.030; Figure 3G). The consumption of decaf did not alter behavioral performance compared to water-consuming male mice (*n* = 8 per group) and most of the differences identified between water-consuming and caff-consuming male mice were also observed between male mice consuming caff and decaf (Figure 2 and Figure 3).

With respect to female mice, the consumption of caff compared to water only (*n* = 8 per group), did not modify either weight (*p* = 0.141; Figure 2H) or locomotion (*p* = 0.580; Figure 2I) or sensorimotor coordination (*p* = 0.793; Figure 2J) or anxiety in the open field (*p* = 0.129; Figure 2K) or in the elevated plus maze (*p* = 0.305; Figure 2L) or working memory (*p* = 0.805; Figure 2M); however, hierarchy in the tube test was modified showing an increased dominance of female mice drinking caff (*p* = 0.046; Figure 2N). There were no significant alterations of despair behavior (*p* = 0.607; Figure 3H) or of resilience to adversity in the forced swimming test (*p* = 0.064; Figure 3I), but there was a greater tendency for selfcare (*p* = 0.021; Figure 3J) and motivation in the splash test (*p* = 0.003; Figure 3K). In contrast to male mice, the intake of caff did not modify the sociability pattern of female mice (*p* = 0.638; Figure 3L). Finally, caff intake caused inconsistent effects on wellbeing of female mice, as gauged by the lower latency to begin nest building (*p* = 0.049 vs. water, *n* = 8; Figure 3M) but similar burrowing in the marble burying test (*p* = 0.987; Figure 3N). The consumption of decaf did not alter behavioral performance compared to water-consuming female mice (*n* = 8 per group); however, apart from the impact on hierarchy, the differences identified between water-consuming and caff-consuming female mice were not observed between female mice consuming caff and decaf (Figure 2 and Figure 3).

In summary, only caffeinated rather than decaffeinated coffee modified the behavior of mice, an effect different in males and females: males become more sociable and with increased wellbeing albeit with a tendency to become more anxious, whereas the main effects of the intake of caffeinated coffee in females is to increase dominance and selfcare. A post-hoc two-way ANOVA revealed sex-differences in the effects of caffeine (*p* values for interaction between the effect of caffeinated coffee and sex) on the hierarchy in the tube test (*p* = 0.044; dominance), in the time socializing (*p* = 0.044) and in the latency to grooming (*p* = 0.048; selfcare), but not in the marble burying (*p* = 0.089; wellbeing). 

### 3.2. Alterations of Brain Antioxidant Status

There is ample evidence that the excessive accumulation of radical species is associated with brain diseases defined symptomatologically by behavioral alterations [53]. Moreover, there is compelling evidence linking the consumption of coffee [54] as well as major coffee constituents such as polyphenols [18,19] to the control of antioxidant defenses and to the excessive accumulation of radical species. Thus, we first aimed to test if the consumption of caff and decaf might affect readouts of oxidative status and antioxidant systems in the frontal cortex (*n* = 8 per group, except when otherwise specified), as a possible explanation for the behavioral effects of caff rather than of decaf. 

**Figure 4:**
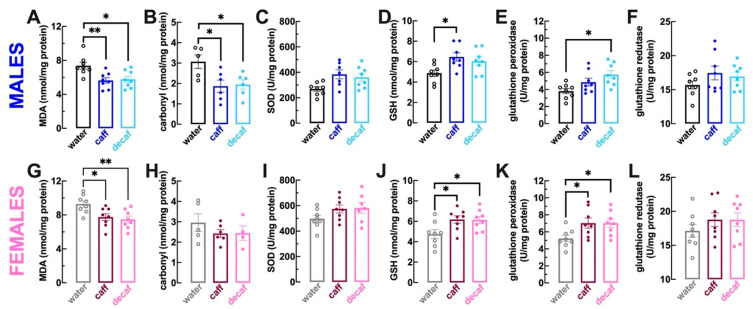
**Caffeinated or decaffeinated coffee decreases oxidative stress and bolsters antioxidant status in the frontal cortex of both male and female mice.** Adult mice (12 weeks old) either males (upper row in blue) or females (bottom row in pink) had free access to either water (black and grey bars), caffeinated coffee (caff, 1 g/L; in dark blue or dark pink) or decaffeinated coffee (decaf, 1 g/L; in light blue or pink) during the night period for at least 3 weeks before beginning behavioral analysis as described in Figure 1, which was followed by the sacrifice of the animals for collection of brain tissue. Compared to control water-consuming mice, caff and decaf decreased lipid peroxidation (levels of malonaldehyde, MDA) in both males (**A**) and females (**G**) as well as protein oxidation (levels of carbonyl groups) in males (**B**) but not in females (**H**). Caff and decaf did not alter the activity of superoxide dismutase (SOD) in males (**C**) or females (**I**) but increased the levels of glutathione in males (**D**) and females (**J**) as well as the activity of glutathione peroxidase in males (**E**) and females (**K**) without altering the activity of glutathione reductase in males (**F**) and females (**L**). Data are mean ± SEM; *n* = 5–8 mice per group. * *p* < 0.05, ** *p* < 0.01 between indicated groups using a one-way ANOVA followed by a Tukey’s multiple comparison test.

We first quantified malondialdehyde (MDA) as a lipid peroxidation marker [55] and protein carbonyl groups as a protein oxidation marker [56]. Compared to mice consuming only water, the regular intake of caff or of decaf decreased the levels of MDA both in male mice (*p* = 0.008 for caff, *p* = 0.014 for decaf; Figure 4A) as well as in female mice (*p* = 0.029 for caff, *p* = 0.009 for decaf; Figure 4G); likewise, the levels of carbonyl groups were also decreased in male mice consuming caff or decaf (*p* = 0.028 for caff, *p* = 0.041 for decaf vs. water, *n* = 5–7; Figure 4B). In female mice, the values failed to reach statistical significant probably due to the loss of some samples during the quantification procedure (water: 2.96 ± 0.43 nmol/mg protein, *n* = 5; caff: 2.43 ± 0.19 nmol/mg protein, *n* = 6, *p* = 0.463 vs. water; decaf: 2.96 ± 0.43 nmol/mg protein, *n* = 4, *p* = 0.545 vs. water; Figure 4H).

Although we did not find significant alterations of the activity of superoxide dismutase (SOD), a major antioxidant system quenching oxygen radicals [57], in either male or female mice consuming either caff (males: *p* = 0.054 vs. water, Figure 4C; females: *p* = 0.241 vs. water, Figure 4I) or decaf (males: *p* = 0.068 vs. water, Figure 4C; females: *p* = 0.209 vs. water, Figure 4I), the intake of caff increased the levels of the main soluble antioxidant, reduced glutathione (GSH; [53]) in both male (*p* = 0.019 vs. water; Figure 4D) and female mice (*p* = 0.038 vs. water; Figure 4J). The levels of GSH were also enhanced in female mice consuming decaf (*p* = 0.045 vs. water; Figure 4J) but they did not reach statistical significance in male mice (*p* = 0.083 vs. water; Figure 4D). The activity of glutathione peroxidase was also increased upon regular consumption of decaf in both male (*p* = 0.009 vs. water; Figure 4E) and female mice (*p* = 0.049 vs. water; Figure 4K), whereas the intake of caff also increased the activity of glutathione peroxidase in female mice (*p* = 0.047 vs. water; Figure 4K) and it did not reach statistical significance in male mice (*p* = 0.196 vs. water; Figure 4E). Finally, the activity of glutathione reductase was not affected by the intake of caff or decaf either in male mice (caff: *p* = 0.259 vs. water, Figure 4C; decaf: *p* = 0.495 vs. water, Figure 4F) or female mice (caff: *p* = 0.483 vs. water, Figure 4C; decaf: *p* = 0.492 vs. water, Figure 4L).

In summary, both caffeinated and decaffeinated coffee tended to cause a parallel reduction of the oxidative status of lipids and proteins and to increase the levels of antioxidants in the frontal cortex. This parallel effect of caff and decaf on oxidative status contrasts with the different behavioral effects of caff and decaf, making it unlikely that the control of oxidative stress might underlie the behavioral effects of caff. Finally, it is worth noting that female mice tended to display an oxidative status as well as antioxidant levels in their frontal cortex larger than that found in males, reenforcing the sex difference in the status and impact of oxidative stress (e.g., [58,59]).

### 3.3. Alterations of Adenosine Neuromodulation

A main homeostatic controller directly affected by coffee intake is the adenosine neuromodulation system, in view of the evidence showing that the only targets of caffeine in non-toxic levels are adenosine receptors [9,10]. Adenosine is tonically present to homeostatically decrease the noise in neuronal circuits through the activation of inhibitory A_1_ receptors (A_1_R), which activation has been linked to mood control [60] and neuroprotection [61]. Thus, we next investigated if the differential behavioral effects of caff and decaf would be associated with a different impact of caff and decaf on the density and function of A_1_R (*n* = 8 per group) in some brain areas associated with mood control such as the hippocampus and striatum [25,27,28].

**Figure 5:**
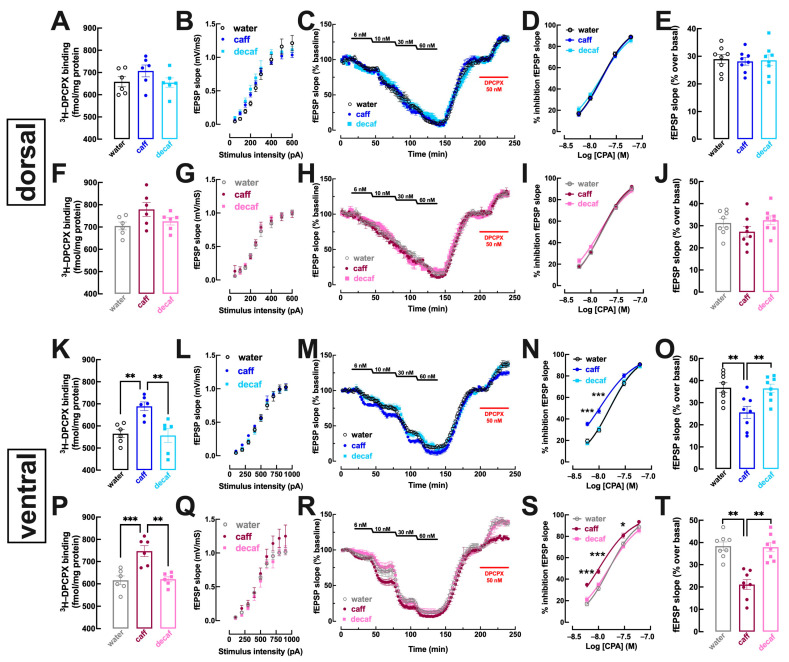
**Adenosine A_1_ receptor density and function are increased by caffeinated but not decaffeinated coffee selectively in the ventral but not dorsal hippocampus in both male and female mice**. Adult mice (12 weeks old) either males (upper and third row in blue) or females (second and bottom row in pink) had free access to either water (black and grey bars), caffeinated coffee (caff, 1 g/L; in dark blue for males or dark pink for females) or decaffeinated coffee (decaf, 1 g/L; in light blue for males or pink for females) during the night period for at 3 weeks before beginning behavioral analysis as described in Figure 1 followed by sacrifice and preparation of membranes or slices (400 μm thick) of the dorsal hippocampus (top two rows) or of the ventral hippocampus (bottom two rows). In the dorsal hippocampus (**A**–**J**), neither caff nor decaf modified the density of adenosine A_1_ receptors (A_1_R) assessed by ^3^H-DPCPX binding to whole membranes (**A**,**F**), nor synaptic transmission ((**B**,**G**), assessed by input/output curves in slices) nor the inhibitory effect on synaptic transmission (measured by the slope of evoked excitatory post-synaptic potentials, fEPSP, recorded in CA1 synapses upon stimulation of afferent Schaffer fibers) of cumulative concentrations of the selective A_1_R agonist CPA (**C**,**D**,**H**,**I**) nor the disinhibitory effect of DPCPX on synaptic transmission that indirectly inform on the levels of extracellular adenosine in synapses (**C**,**E**,**H**,**J**). In contrast, in the ventral hippocampus (**K**–**T**), caff increased the density of A_1_R (**K**,**P**), increased the potency of CPA to inhibit synaptic transmission (**M**,**N**,**R**,**S**) and decreased the levels of extracellular synaptic adenosine (**M**,**O**,**R**,**T**), whereas decaf was devoid of effects on A_1_R function (**K**–**T**). Data are mean ± SEM; *n* = 7–8 mice per group. * *p* < 0.05, ** *p* < 0.01, *** *p* < 0.001 between indicated groups using a one-way ANOVA followed by a Tukey’s multiple comparison test.

In the dorsal hippocampus, mostly associated with processing memory traits [25] that were not significantly affected by the intake of caff, there was no modification of the binding density of the A_1_R selective antagonist ^3^H-DPCPX in mice consuming either caff (males: *p* = 0.339, Figure 5A; females: *p* = 0.083, Figure 5F) or decaf (males: *p* = 0.991, Figure 5A; females: *p* = 0.981, Figure 5F). Likewise, there were no evident modifications of the synaptic responsiveness in dorso-hippocampal synapses in males (Figure 5B) or females (Figure 5G) and neither the inhibitory effect of the A_1_R agonist CPA (Figure 5C,D,H,I) nor the disinhibitory effect of the A_1_R antagonist (Figure 5C,E,H,J) were modified in either male (Figure 5C–E) or female mice (Figure 5H–J) consuming either caff or decaf.

This lack of modification of the adenosine modulation in the dorsal hippocampus upon caff consumption contrasted with the modifications observed in the ventral hippocampus, a brain region involved in processing mood-related information [28]. Thus, there was an increased density of A_1_R in whole membranes of the ventral hippocampus of mice consuming caff both males (*p* = 0.006; Figure 5K) and females (*p* = 0.001; Figure 5P), whereas there were no significant changes of A_1_R density in mice consuming decaf in either males (*p* = 0.968; Figure 5K) or females (*p* = 0.981; Figure 5P). Accordingly, CPA caused a greater inhibition of synaptic transmission in mice consuming caff, either males (water: EC_50_ = 16.38 nM, 95% confidence interval 13.93–18.83 nM vs. caff: EC_50_ = 10.49 nM, 95% confidence interval 8.68–12.29 nM; *p* = 0.001; Figure 5M,N) or females (water: EC_50_ = 16.33 nM, 95% confidence interval 14.67–17.98 nM vs. caff: EC_50_ = 10.36 nM, 95% confidence interval 8.92–11.80 nM; *p* < 0.001; Figure 5R,S), whereas there was no modification of CPA-induced inhibition of synaptic transmission in mice consuming decaf, either males (EC_50_ = 16.21 nM, 95% confidence interval 13.95–18.47 nM; *p* = 0.990 vs. water; Figure 5M,N) or females (EC_50_ = 16.13 nM, 95% confidence interval 13.74–18.51 nM; *p* = 0.983 vs. water; Figure 5R,S). Additionally, the intake of caff also altered the tonic activation of A_1_R by endogenous adenosine in the ventral hippocampus, as concluded from the lower disinhibitory effect of DPCPX in both caff-consuming males (*p* = 0.007 vs. water; Figure 5M,O) and females (*p* < 0.001 vs. water; Figure 5R,T). In contrast, the intake of decaf did not significantly modify the disinhibitory effect of DPCPX in both males (*p* = 0.995 vs. water; Figure 5M,O) and females (*p* = 0.984 vs. water; Figure 5R,T).

**Figure 6:**
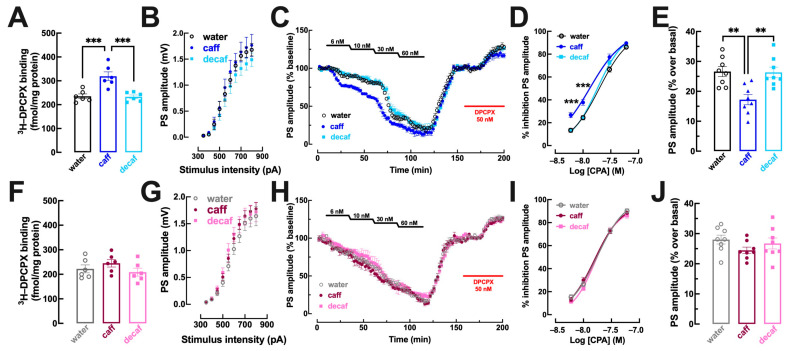
**Adenosine A_1_ receptor density and function are increased by caffeinated but not decaffeinated coffee in the striatum selectively in male but not in female mice**. Adult mice (12 weeks old) either males (upper row in blue) or females (bottom row in pink) had free access to either water (black and grey bars), caffeinated coffee (caff, 1 g/L; in dark blue for males or dark pink for females) or decaffeinated coffee (decaf, 1 g/L; in light blue for males or pink for females) during the night period for at least 3 weeks before beginning behavioral analysis as described in Figure 1 followed by sacrifice and preparation of striatal membranes or cortico-striatal slices (400 μm thick). In males mice (**A**–**E**), caff but not decaf increased the density of adenosine A_1_ receptors (A_1_R) assessed by ^3^H-DPCPX binding in whole striatal membranes (**A**), increased the inhibitory effect on synaptic transmission (measured by the amplitude of evoked population spike, PS, recorded in the dorsomedial striatum upon stimulation of afferent cortical fibers) of cumulative concentrations of the selective A_1_R agonist CPA (**C**,**D**) and decreased the disinhibitory effect of DPCPX on synaptic transmission that indirectly inform on the levels of extracellular adenosine in synapses (**C**,**E**). In contrast, in females (**F**–**J**) caff as well as decaf did not modify either A_1_R density (**F**) or the potency of CPA to inhibit synaptic transmission (**G**,**I**) or the levels of extracellular synaptic adenosine (**H**,**J**). Data are mean ± SEM; *n* = 7–8 mice per group. ** *p* < 0.01, *** *p* < 0.001 between indicated groups using a one-way ANOVA followed by a Tukey’s multiple comparison test.

In the striatum, which in tightly associated with processing of anhedonic and motivational behaviors [27] in particular in its trigger—corticostriatal synapses [62], there were also sex-dependent modifications of the adenosine modulation system upon consumption of caff whereas decaf did not cause any adaptive alterations of the adenosine modulation system in the striatum (Figure 6). Thus, there was an increased density of A_1_R in whole membranes of the striatum of mice consuming caff in males (*p* = 0.001; Figure 6A) but not in females (*p* = 0.580; Figure 6F), whereas there were no significant changes of A_1_R density in mice consuming decaffeinated coffee in either males (*p* = 0.992; Figure 6A) or females (*p* = 0.828; Figure 6F). Accordingly, CPA caused a greater inhibition of synaptic transmission in mice consuming caff in males (water: EC_50_ = 19.80 nM, 95% confidence interval 17.37–22.23 nM vs. caff: EC_50_ = 13.54 nM, 95% confidence interval 10.97–16.11 nM; *p* = 0.001; Figure 6C,D) but not in females (water: EC_50_ = 17.32 nM, 95% confidence interval 15.31–19.33 nM vs. caff: EC_50_ = 17.21, 95% confidence interval 15.04–19.381 nM; *p* = 0.996; Figure 6H,I), whereas there was no modification of CPA-induced inhibition of synaptic transmission in mice consuming decaf, either males (EC_50_ = 18.27 nM, 95% confidence interval 16.29–20.25 nM; *p* = 0.530 vs. water; Figure 6C,D) or females (EC_50_ = 18.79 nM, 95% confidence interval 16.40–21.18 nM; *p* = 0.512 vs. water; Figure 6H,I). Additionally, the intake of caff also altered the tonic activation of A_1_R by endogenous adenosine in the striatum of male mice, as concluded from the lower disinhibitory effect of DPCPX in males (*p* = 0.002 vs. water; Figure 6C,E) but not in females (*p* = 0.231 vs. water; Figure 6H,J). In contrast, the intake of decaf did not significantly modify the disinhibitory effect of DPCPX in both males (*p* = 0.993 vs. water; Figure 6C,E) and females (*p* = 0.813 vs. water; Figure 6H,J).

## 4. Discussion

The present study provides evidence indicating that the regular intake of moderate doses of coffee ameliorates emotional behavior and wellbeing in adult male and female mice. The amount consumed of either caffeinated or decaffeinated coffee during the over 3 weeks period (only during the night, i.e., active period) were indistinguishable from water-consumption. Coffee has a bitter taste normally aversive to animals [63] and overcoming this hurdle for consumption is in itself suggestive of perceived benefits that override this presumed taste barrier, which is signaled through mesolimbic dopamine release [64], a pathway also involved in reward and anticipated pleasurable experiences [65]. Thus, this apparently surprising adherence of mice to the consumption of these bitter coffee mixtures may well be derived from the presently established increased in wellbeing afforded by the regular intake of coffee.

A mood heightening effect was observed with caffeinated coffee, but not with decaffeinated coffee. Although the chemical composition of both beverages as well as the levels and pharmacokinetics of different coffee components were not determined in this naturalistic-like study, the striking behavioral difference between caffeinated and decaffeinated coffee is strongly suggestive of ascribing a major mood-related behavioral effect to caffeine. Our previous investigation of the behavioral effects of caffeine showed that adult male mice drinking water-solubilized caffeine (0.3 g/L) for 2 weeks displayed a locomotor and memory pattern indistinguishable from control mice (drinking water) [14]. Our previous studies of the impact of caffeine also did not find an altered immobility in the forced swimming test in adult male mice [14,33], as now also observed upon intake of caffeinated coffee (Figure 3). However, the presently more detailed behavioral study highlights an ability of the regular intake of caffeinated coffee to selectively ameliorate emotional behavior without effects on locomotion or memory. Accordingly, previous studies showed that the regular intake of caffeine prevents emotional dysfunction associated with repeated stress in mice [33,66,67,68,69] involving the antagonism of adenosine A_2A_ receptors [33,70] and the control of neuronal activity in stress-related areas [70,71]. Accordingly, human epidemiological studies showed that the regular consumption of coffee and dietary caffeine has a protective effect against the development of depression [72,73,74,75] an effect most evident with caffeinated rather than decaffeinated coffee [74,76] (but see [77]). Furthermore, the regular intake of coffee attenuates the perception of stress [78,79,80,81] and mitigates emotional responses to negative or stressful situations [82,83,84]. Thus, the overall evidence converges to support the contention that the regular intake of coffee normalizes mood changes and prevents mood dysfunction. However, the impact on wellbeing is less clear. In fact, there is a notorious lack of studies judging the impact of coffee intake on wellbeing and the few available are inconsistent: some studies failed to find substantive associations between coffee intake and psychological wellbeing over up to 20 years of follow-up in a large-scale cohort of midlife and older women [85] and with mental wellbeing in midlife women [86]; in contrast, other studies report a positive association between psychological wellbeing with caffeinated coffee and total caffeine intake [87,88] and identified caffeinated beverages as contributors to the perception of subjective wellbeing [89]. Our present evidence linking the intake of caffeinated coffee to a bolstering of wellbeing in mice is hoped to contribute to counteracting the bulk of studies on the intake of caffeinated products, which mostly focuses on caffeine use disorder, caffeine withdrawal symptoms and perceived harm (e.g., [90,91,92]) rather than on wellbeing.

An effect of a regular intake of caffeinated coffee on wellbeing is further rooted on the previous observation that the regular intake of moderate doses of caffeine alters brain functional connectivity in humans [15,16] and modifies synaptic and metabolic processes [13] in particular in synapses [14] of the hippocampus of male mice. We now observed that the mood-related behavioral effects of caffeinated coffee were associated with an alteration of the adenosine modulation system selectively in brain regions involved in processing mood information (ventral hippocampus and striatum) without alterations in brain regions primarily processing reference memory (dorsal hippocampus) [25,27,28]. We found an increased density of adenosine A_1_ receptors (A_1_R) coupled to an increased potency of an A_1_R agonist to inhibit excitatory synaptic transmission. This is in striking agreement with previous observations that an increased signaling through A_1_R inhibit depressive-like behavior [60,93,94], although it is still unclear where and how an increased A_1_R function might impact the resilience to stress [95]. In parallel with this increased potential of A_1_R, we observed that the intake of caffeinated coffee decreased the tonic inhibition by endogenous adenosine, a process that occurred selectively in brain areas processing mood (ventral hippocampus and striatum) but not in the dorsal hippocampus. Basal adenosine levels are mostly controlled by the activity of nucleoside transporters [96], which is in agreement with previous reports that tinkering with the activity of equilibrative nucleoside transporters impacts on the emotional status of rodents [97,98], namely controlling the motivational drive of the transition from habitual to goal-directed reward-seeking behaviors [99]. This effect involves the activation of astrocytes in the dorsomedial striatum [99] in accordance with the key role of equilibrative nucleoside transporters to control astrocytes [100,101] that are increasingly implicated in the control emotional status and depression [102,103,104]. This raises the novel untested hypothesis that the behavioral effects of coffee might involve a control of astrocytes [104] and astrocyte-to-neuron communication [105], with a dampening of A_1_R-mediated tonic inhibition being tentatively associated with increased activity and functional connectivity in brain areas processing emotional status in human volunteers [15,16]. Future studies will be required to confirm this working hypothesis and to establish causative evidence to define the relative contribution of brain-mediated actions and eventual peripheral effects of caffeinated coffee indirectly influencing mood and wellbeing.

The selective mood ameliorating effect of caffeinated rather than decaffeinated coffee and its association with alterations of the adenosine modulation system selectively in areas mainly involved in emotional processing, makes it less likely that other prominent coffee components such as chlorogenic acids may be major players in the mood ameliorating effects of caffeinated coffee. Accordingly, clinical trials in human volunteers failed to reveal clear effects of chlorogenic acid-rich beverages on mood and emotional related domains of behavioral performance [106,107,108]. Furthermore, the main mechanism argued to be engaged by chlorogenic acids—bolstering of antioxidant status—was documented in the present study to occur in the frontal cortex of mice upon consumption of both caffeinated as well as decaffeinated coffee, in contrast to the selective mood enhancing effects of caffeinated rather than decaffeinated coffee. This ability of the regular intake of both caffeinated and decaffeinated to decrease markers of oxidative stress and bolster antioxidant systems in the frontal cortex, although likely unrelated to increased wellbeing, may be relevant to understand the parallel effects of caffeinated and decaffeinated coffee in decreasing the susceptibility to brain diseases and to increase healthspan upon aging [21,22,23], thanks to a major contribution of beneficial effects of chlorogenic acids controlling antioxidant status [18,19] coupled with caffeine-mediated effects involving the rebalancing of the adenosine modulation system [12,109]. In fact, a major limitation of this study is that it failed to assess the levels of coffee-containing bioactive substances in the beverages and in the animals and did not monitor the individual intake of coffees. Additionally, the extrapolation of animal behavior to humans needs to be considered with care.

Although we globally concluded that the intake of caffeinated coffee ameliorates mood and wellbeing, we noted some striking differences between males and females. In males, which are most often used experimentally, the intake of caffeinated coffee increased the propensity to engage in non-essential “luxury” behaviors that are often used as indicators of positive wellbeing, such as nest building or burrowing activity in the marble burying test. Additionally, male mice displayed more sustained motivated behaviors, typified by a more sustained grooming in the splash test, greater resilience to adversity such as increased struggling in the forced swimming test and increased sociability. Indeed, the impact of caffeine on sociability in rats is dependent on the levels of testosterone [110]. However, the involvement of the adenosine modulation system on sociability is less clear, with increased levels of adenosine (through ketogenic diet, [111]) increasing sociability [112] and A_2A_ receptor blockade increasing sociability [113], whereas ambient caffeine decrease appetence to the shoal (sociability) in adult zebrafish [114]. Some previous studies in male rodents also associated the adenosine modulation system to the control of motivation and selection of goal-directed actions [115,116,117], effort-related behavior [35,118] and fatigue [119,120], all aspects matching the presently reported selective increase of motivational and resilient-related behaviors upon regular consumption of caffeinated coffee by male mice. Strikingly, the impact of the regular intake of caffeinated coffee in female mice was somewhat different from that observed in males. A main difference was the increase in dominant behavior by female mice, which was not observed in males. Also, female mice drinking caffeinated coffee displayed an increase of selfcare unlike males. This partially superimposable behavioral pattern with differences observed between males and females was associated with a superimposable modification of the adenosine modulation system in the ventral (but not dorsal) hippocampus but with a different alteration of the adenosine modulation system in corticostriatal synapses, where alterations were observed in males but not in females drinking caffeinated coffee. Accordingly, there are sex differences in the availability of brain A_1_R [121] and in the release of adenosine in different brain areas [122]. Previous studies had related gender differences in subjective and physiological responses to caffeine to the impact of steroid hormones [123], a contention reenforced by the observation that gender differences in response to caffeine only emerge after puberty [124]. Furthermore, estradiol modifies caffeine neuroprotection in models of Parkinson’s disease [125] and modifies the in vitro cellular effects of caffeine [126]. Conversely, coffee and caffeine can modify the levels of endogenous sex hormones [127,128] and of sex hormone-binding globulin [129,130,131] to differently affect behavior in males and females. Overall, this intricate interplay between sex hormones and coffee/caffeine intake and effects justifies the presently reported tentative identification of sexual dimorphisms in the effects of caffeinated coffee, which prompt considering more detailed investigations on the effects of coffee, caffeine and of the adenosine modulation systems in females that are clearly understudied.

## 5. Conclusions

In conclusion, the present study concluded that the intake of caffeinated, but not decaffeinated, coffee bolsters mood and wellbeing in both males and females, with ameliorations seen in different domains in the two sexes. Furthermore, alterations were observed in the adenosine modulation system in brain areas associated with mood processing, prompting the suggestion that caffeine may play a central role. It is hoped that future studies may confirm in healthy humans the present findings of increased mood and wellbeing upon caffeinated coffee consumption in mice.

## Data Availability

The data that support the findings of this study are available from the corresponding author upon reasonable request.

## List of Abbreviations

A_1_R: adenosine A_1_ receptor; aCSF: artificial cerebrospinal fluid; BCA: bicinchoninic acid; caff: caffeinated coffee; CPA: N^6^-cyclopentyladenosine; decaf: decaffeinated coffee, DPCPX: 1,3-dipropyl-8-cyclopentylxanthine; fEPSP: field excitatory post-synaptic potentials; GSH: glutathione; I/O: input/output curve; MDA: malonaldehyde; PS: population spike; SOD: superoxide dismutase.
